# Risk factors for mortality among neonates admitted to a special care unit in a low-resource setting

**DOI:** 10.1186/s12884-020-03429-2

**Published:** 2020-11-23

**Authors:** Francesco Cavallin, Teresa Bonasia, Desalegn Abebe Yimer, Fabio Manenti, Giovanni Putoto, Daniele Trevisanuto

**Affiliations:** 1 Independent statistician, Solagna, Italy; 2 Doctors with Africa CUAMM, Wolisso, Ethiopia; 3 Department of Neonatal and Pediatric Critical Care, University of Verona, Verona, Italy; 4 Public Health Coordinator, St. Luke Catholic Hospital, Wolisso, Ethiopia; 5 Doctors with Africa CUAMM, Padua, Italy; 6 Department of Woman’s and Child’s Health, University of Padua, Via Giustiniani 3, 35128 Padua, Italy

**Keywords:** Low-resource setting, Mortality, Neonates, Risk factors

## Abstract

**Background:**

Although under-5 mortality has decreased in the last two decades, neonatal mortality remains a global health challenge. Despite achieving notable progress, Ethiopia has still one of the highest neonatal mortality rates worldwide. We aimed to assess the risk factors for mortality among neonates admitted to a special care unit in a referral hospital in rural Ethiopia.

**Methods:**

This was a retrospective observational study including all 4182 neonates admitted to the special care unit of the St. Luke Wolisso Hospital (Ethiopia) from January 2014 to December 2017. Data were retrieved from hospital charts and entered in an anonymized dataset. A logistic regression model was applied to identify predictors of mortality and effect sizes were expressed as odds ratios with 95% confidence intervals.

**Results:**

Proportion of deaths was 17% (709/4182 neonates). Neonates referred from other health facilities or home (odds ratio 1.52, 95% confidence interval 1.21 to 1.91), moderate hypothermia at admission (odds ratio 1.53, 95% confidence interval 1.09 to 2.15) and diagnosis of late-onset sepsis (odds ratio 1.63, 95% confidence interval 1.12 to 2.36), low birthweight (odds ratio 2.48, 95% confidence interval 2.00 to 3.09), very low birthweight (odds ratio 11.71, 95% confidence interval 8.63 to 15.94), extremely low birthweight (odds ratio 76.04, 95% confidence interval 28.54 to 263.82), intrapartum-related complications (odds ratio 4.69, 95% confidence interval 3.55 to 6.20), meconium aspiration syndrome (odds ratio 2.34, 95% confidence interval 1.15 to 4.43), respiratory distress (odds ratio 2.25, 95% confidence interval 1.72 to 2.95), other infections (odds ratio 1.92, 95% confidence interval 1.31 to 2.81) or malformations (odds ratio 2.32, 95% confidence interval 1.49 to 3.57) were associated with increased mortality. Being admitted in 2017 vs. 2014 (odds ratio 0.71, 95% confidence interval 0.52 to 0.97), and older age at admission (odds ratio 0.95, 95% confidence interval 0.93 to 0.97) were associated with decreased likelihood of mortality.

**Conclusions:**

The majority of neonatal deaths was associated with preventable and treatable conditions. Education on neonatal resuscitation and postnatal management, and the introduction of an on-call doctor for high-risk deliveries might have contributed to the reduction in neonatal mortality over time.

## Background

Although under-5 mortality has decreased in the last two decades, neonatal mortality remains a global health challenge [1]. Worldwide, 2.4 million neonates die every year, a third of them within the first day of life [1, 2]. The vast majority of neonatal deaths occur in sub-Saharan Africa and Southern Asia [3, 4]. The main causes of neonatal mortality are prematurity (35%), intrapartum-related events (24%) and infections (15%) [5].

Despite achieving notable progress in neonatal mortality during 1999–2018 (from 49.5 deaths to 28.1 deaths per 1000 live births), Ethiopia has still one of the highest neonatal mortality rates worldwide [6]. Infant mortality is considered a standard indicator for the assessment of a country health status [7] and warrants for continuous research on interventions to achieve the United Nations Sustainable Development Goals (SDGs) [8]. Many neonatal deaths can be prevented with feasible and low-cost interventions [9]. Knowledge of risk factors for mortality can be useful in identifying areas of intervention and planning appropriate strategies to improve neonatal prognosis [10].

Previous studies showed varying figures (neonatal mortality 5.7–16.5%) and heterogeneous risk factors for neonatal mortality (i.e. lack of antenatal care, outborn infants, home delivery, cesarean section, multiple birth, low birth weight, inability at cry at birth, perinatal asphyxia, need for resuscitation at birth, and respiratory distress syndrome) across different areas of Ethiopia [11–13], thus requiring further investigation to expand the knowledge and support the identification of appropriate interventions to reduce neonatal mortality [14]. This study aimed to assess the risk factors for mortality among neonates admitted to a special care unit in a referral hospital in rural Ethiopia.

## Methods

### Study design

This was a retrospective observational study on risk factors for mortality among neonates admitted to the special care unit of the St. Luke Wolisso Hospital (Ethiopia) from January 2014 to December 2017. The study was conducted according to Helsinki Declaration principles, and was approved by the Ethical Review Committee of St Luke Catholic Hospital and College of Nursing and Midwifery (ref. 245/2020), which waived the need for patient written consents, given the retrospective nature of the study and the use of anonymized data.

### Setting

The study was conducted at the special care unit of the St. Luke Wolisso Hospital (Ethiopia), where around 3500 deliveries occur every year. This is a referral, private, non-profit hospital located in Wolisso town, which is the capital of the Southwest Shoa Zone in the Oromiya region. The area has a population of about 1.1 million inhabitants and is served by 81 health facilities (including only one hospital). At St. Luke Wolisso Hospital, midwives were responsible for maternal and neonatal management at delivery. Medical transport system was not available in the area and outborn infants were brought to the hospital by their families. The special care unit consisted in one room with 10 beds and was cared for by two daytime nurses and one nighttime nurse. Phototherapy, intravenous therapies and oxygen supplementation (but not equipment for respiratory support) were offered. Availability of pulse oxymeter was limited. During the study period, education on neonatal resuscitation (Helping Babies Breathe program) and courses on postnatal management were offered to midwives and nurses, and the presence of an on-call doctor for high-risk deliveries was introduced.

### Patients

All neonates admitted to the special care unit at the St. Luke Wolisso Hospital between 2014 and 2017 were included in the study. There were no exclusion criteria.

### Data collection

All data were retrospectively retrieved from hospital charts and entered in an anonymized dataset. Data included neonatal characteristics (i.e. age, sex, weight), admission information (neonatal temperature, outborn/inborn, mode of delivery, main diagnoses), length of stay and outcome. Clinical definitions are summarized in Table 1 [15–21].

**Table 1:** Clinical definitions

Diagnosis at admission	Definition
Sepsis	Maternal history of fever or prolonged rupture of membranes > 18 h, and/or neurologic findings (apnea, convulsions, unconsciousness), and/or moderate hypothermia (< 36 °C) or hyperthermia (> 37.5 °C), and/or breastfeeding difficulties [15–18] within 72 h (early onset) or later (late onset).
Other infections	Skin infections, abscesses, genital-urinary infections, pneumonia.
Low birthweight	Birthweight below 2500 g [16]. Sub-groups in the manuscript were: -LBW (low birthweight, 1500–2499 g), -VLBW (very low birth weight, 1000–1499 g), -ELBW (extremely low birth weight, < 1000 g).
Intrapartum-related complications	Failure to initiate spontaneous regular respirations after birth and/or 5-min Apgar score less than 7 and/or Sarnat & Sarnat stage 2 or more at admission [10, 15, 19].
Meconium aspiration syndrome (MAS)	Presence of respiratory distress in infants born through meconium-stained amniotic fluid [20].
Respiratory distress	Signs of respiratory insufficiency (tachypnea, dyspnea, and grunting) and need for oxygen supplementation for more than 2 days [21].
Transient tachypnea of the newborn	Presence of tachypnea and need for oxygen supplementation within 2 days of life [21].
Malformations	Diagnosis was based on clinical examination and/or radiological investigation (i.e. X-rays and Doppler ultrasound). Magnetic resonance imaging (MRI), computer tomography (CT), chromosome analysis and chromosome microarray were not available in the hospital.
Neonatal hypothermia at admission	Mild hypothermia: 36–36.4 °C [16]. Moderate hypothermia: neonatal temperature 32–35.9 °C [16]. Severe hypothermia: neonatal temperature < 32 °C [16].
Neonatal hyperthermia at admission	Neonatal temperature > 37.5 °C [16].

### Statistical analysis

Continuous variables were expressed as median and interquartile range (IQR), and categorical variables as number and percentage. Association between categorical variables was evaluated with Chi Square test. A logistic regression model was applied to identify predictors of mortality among clinically relevant factors (year of admission, mode of delivery, birthplace, age and temperature at admission, sex, birth weight, early-onset sepsis, late-onset sepsis, intrapartum-related complications, meconium aspiration syndrome, respiratory distress, transient tachypnea of the newborn, other infections, malformations). Multicollinearity was assessed using variance inflation factor (VIF), with values > 4 suggesting further investigation and values > 10 indicating need for correction for multicollinearity. Estimated effects from the model were expressed as odds ratios (OR) with 95% confidence intervals (CI). Trends over time of the prevalence of prognostic factors were investigated with linear and logistic regression models where time was modeled with linear and quadratic terms. All tests were 2-sided and a *p*-value less than 0.05 was considered statistically significant. Statistical analysis was performed using R 3.5 software (R Foundation for Statistical Computing, Vienna, Austria) [22].

## Results

### Patients

Overall, 4182 neonates (2424 males and 1758 females) were admitted to the special care unit of the St. Luke Wolisso Hospital (Ethiopia) from January 2014 to December 2017. Neonatal characteristics are reported in Table 2. Median age at admission was 1 day (IQR 1–4). Almost half of neonates were outborn (1577/3521, 44.8%), while the information was not available in 661 neonates. Among 1179 inborn neonates who were admitted at day of birth, 379 (32%) had moderate hypothermia (32–36 °C) and none severe hypothermia (< 32 °C). Moderate hypothermia was not different (*p* = 0.75) in neonates born through vaginal delivery (439/1794, 26.9%) and in those born through caesarean section (109/480, 28.6%). Moderate hypothermia was 24.8% (323/1300) in outborn and 28.3% (442/1560) in inborn neonates (*p* = 0.04).

**Table 2:** Neonatal characteristics at admission

	All neonates	Neonates who survived	Neonates who died
No. of neonates	4182	3473	709
Year of admission:			
2014	692 (16.5)	563 (16.2)	129 (18.2)
2015	1113 (26.6)	916 (26.4)	197 (27.8)
2016	1278 (30.6)	1059 (30.5)	219 (30.9)
2017	1099 (26.3)	935 (26.9)	164 (23.1)
Age at admission, days ^a^	1 (1–4)	1 (1–5)	1- (1, 2)
Sex:			
Male	2424 (58.0)	2006 (57.8)	418 (59.0)
Female	1758 (42.0)	1467 (42.2)	291 (41.0)
Birth weight:			
Normal weight (≥2500 g)	2756 (65.9)	2440 (70.3)	316 (44.6)
Low birth weight (1500–2499 g)	1125 (26.9)	896 (25.8)	229 (32.3)
Very low birth weight (1000–1499 g)	270 (6.5)	133 (3.8)	137 (19.3)
Extremely low birth weight (< 1000 g)	31 (0.7)	4 (0.1)	27 (3.8)
Temperature at admission: ^b^			
< 32 °C	0 (0.0)	(0.0)	0 (0.0)
32–35.9 °C	779 (26.7)	583 (24.0)	196 (40.5)
36–36.4 °C	759 (26.1)	634 (26.1)	125 (25.8)
36.5–37.5 °C	948 (32.5)	822 (33.8)	126 (26.0)
> 37.5 °C	428 (14.7)	391 (16.1)	37 (7.7)
Outborn ^c^	1577 (44.8)	1291 (44.3)	286 (47.0)
Mode of delivery: ^d^			
Vaginal	1794 (73.1)	1422 (71.1)	372 (82.1)
Instrumental	179 (7.3)	158 (7.9)	21 (4.7)
Caesarean section	480 (19.6)	420 (21.0)	60 (13.2)

Data expressed as No. (%) or ^a^ median (IQR). Data not available in ^b^1268, ^c^661 and ^d^1729 neonates

### Diagnosis at admission

The most common diagnoses at admission were birthweight< 2500 g (1,426, 34.1%), early-onset sepsis (787, 18.8%), respiratory distress (644, 15.4%), intrapartum-related complications (595, 14.2%) and late-onset sepsis (540, 12.9%) (Table 3). Median length of stay was 5 days (IQR 4–8). Eight neonates with spina bifida were transferred to another center for surgical treatment.

**Table 3:** Main diagnoses at admission and mortality rate according to diagnosis at admission

	No. of neonates	Deaths
Early-onset sepsis	787 (18.8)	107/787 (13.6)
Late-onset sepsis	540 (12.9)	75/540 (13.9)
Low birth weight (1500–2499 g)	1125 (26.9)	229/1125 (20.4)
Very low birth weight (1000–1499 g)	270 (6.5)	137/270 (50.7)
Extremely low birth weight (< 1000 g)	31 (0.7)	27/31 (87.1)
Intrapartum-related complications	595 (14.2)	173/595 (29.1)
Meconium aspiration syndrome	67 (1.6)	12/67 (17.9)
Respiratory distress	644 (15.4)	146/644 (22.7)
Transient tachypnea of the newborn	129 (3.1)	8/129 (6.2)
Other infections	490 (11.7)	62/490 (12.7)
Malformations	129 (3.1)	36/129 (27.9)
Hypothermia/hyperthermia: ^a^		
Severe hypothermia (< 32 °C)	0 (0.0)	Nil
Moderate hypothermia (32–36 °C)	779 (26.7)	125/759 (16.5)
Mild hypothermia (36–36.4 °C)	759 (26.1)	196/779 (25.2)
Hyperthermia (> 37.5 °C)	428 (14.7)	37/428 (8.6)

Data expressed as No. (%). ^a^ Temperature at admission was not available in 1268 neonates

### Neonatal mortality

Proportion of deaths was 17% (709/4182) and was highest in ELBW and VLBW neonates (27/31, 87.1%, and 137/270, 50.7%, respectively), and in those with intrapartum-related complications (173/595, 29.1%), malformation (36/129, 27.9%) or respiratory distress (146/644, 22.7%) (Table 3). The majority of deaths (84%) occurred during the first week of admission.

### Predictors of neonatal mortality

At multivariable analysis (Table 4), increased likelihood of mortality was associated with outborn neonates (OR 1.64, 95% CI 1.31 to 2.06) and with diagnosis of late-onset sepsis (OR 1.63, 95% CI 1.12 to 2.36), LBW (OR 2.48, 95% CI 2.00 to 3.09), VLBW (OR 11.71, 95% CI 8.63 to 15.94), ELBW (OR 76.04, 95% CI 28.54 to 263.82), intrapartum-related complications (OR 4.69, 95% CI 3.55 to 6.20), MAS (2.34, 95% CI 1.15 to 4.43), respiratory distress (OR 2.25, 95% CI 1.72 to 2.95), other infections (OR 1.92, 95% CI 1.31 to 2.81) or malformations (OR 2.32 95% CI 1.49 to 3.57). On the other hand, being admitted in 2017 vs. 2014 (OR 0.71, 95% CI 0.52 to 0.97) and older age at admission (OR 0.95, 95% CI 0.93 to 0.97) were associated with decreased likelihood of mortality. Multicollinearity was not present (VIF < 2 for all factors).

**Table 4:** Multivariable analysis of predictors of mortality

	p-value	Odds ratio (95% confidence interval)
Year of admission:	0.03	
2014		Reference
2015		1.00 (0.73 to 1.36)
2016		0.86 (0.63 to 1.16)
2017		0.71 (0.52 to 0.97)
Age at admission, days	0.0003	0.95 (0.93 to 0.97)
Sex:	0.62	
Female		Reference
Male		1.05 (0.86 to 1.28)
Birth weight:	< 0.0001	
Normal weight (≥2500 g)		Reference
Low birth weight (1500–2499 g)		2.48 (2.00 to 3.09)
Very low birth weight (1000–1499 g)		11.71 (8.63 to 15.94)
Extremely low birth weight (< 1000 g)		76.04 (28.54 to 263.82)
Birthplace:	< 0.0001	
Inborn		Reference
Outborn		1.64 (1.31 to 2.06)
Early-onset sepsis:	0.96	
No		Reference
Yes		0.99 (0.75 to 1.31)
Late-onset sepsis:	0.01	
No		Reference
Yes		1.63 (1.12 to 2.36)
Intrapartum-related complications:	< 0.0001	
No		Reference
Yes		4.69 (3.55 to 6.20)
Meconium aspiration syndrome:	0.01	
No		Reference
Yes		2.34 (1.15 to 4.43)
Respiratory distress:	< 0.0001	
No		Reference
Yes		2.25 (1.72 to 2.95)
Transient tachypnea of the newborn:	0.26	
No		Reference
Yes		0.62 (0.25 to 1.33)
Other infections:	0.0007	
No		Reference
Yes		1.93 (1.31 to 2.81)
Malformations:	0.0002	
No		Reference
Yes		2.32 (1.49 to 3.57)

In the subsample of 1994 neonates with complete data on temperature at admission and mode of delivery, moderate hypothermia (32–35.9 °C) at admission was a predictor of mortality (OR 1.53, 95% CI 1.09 to 2.15; p = 0.01), while mild hypothermia (36–36.4 °C), hyperthermia (> 37.5 °C) and mode of delivery were not associated with mortality (p = 0.71, p = 0.98 and p = 0.67, respectively)

In the subsample of 1994 neonates with complete data on temperature at admission and mode of delivery, moderate hypothermia (32–35.9 °C) at admission was a predictor of mortality (OR 1.53, 95% CI 1.09 to 2.15; *p* = 0.01), while mild hypothermia (36–36.4 °C), hyperthermia (> 37.5 °C) and mode of delivery were not associated with mortality (*p* = 0.71, *p* = 0.98 and *p* = 0.67, respectively).

### Change over time in predictors of neonatal mortality

During the study period, there was an increase of outborn neonates (*p* = 0.02) and those with late-onset sepsis (*p* < 0.0001), and a decrease of neonates with moderate hypothermia (*p* = 0.003) or other infections (*p* = 0.0003) (Table 5). The proportion of admissions for LBW (*p* = 0.0001) or malformations (*p* = 0.01) displayed a U-shaped trend, while the proportion of admissions for intrapartum-related complications (*p* = 0.04) or respiratory distress (*p* < 0.0001) displayed an inverted U-shaped trend (Table 5).

**Table 5:** Summary of predictors of mortality during the study period

	2014	2015	2016	2017	*p*-value
No. of neonates	692	1113	1278	1099	–
Age at admission, days ^a^	1 (1–4)	1 (1–4)	1 (1–5)	1 (1–4)	0.77
Low birth weight (1500–2499 g)	202 (29.1)	258 (23.2)	323 (25.3)	342 (31.1)	0.0001 ^b^
Very low birth weight (1000–1499 g)	46 (6.6)	56 (5.0)	82 (6.4)	86 (7.8)	0.09
Extremely low birth weight (< 1000 g)	3 (0.4)	9 (0.8)	11 (0.9)	8 (0.7)	0.56
Outborn	202 (39.1)	401 (45.6)	514 (46.6)	460 (45.1)	0.02 ^b^
Moderate hypothermia (32–35.9 °C)	171 (38.4)	156 (21.6)	218 (28.2)	234 (24.0)	0.003 ^b^
Late-onset sepsis	56 (8.1)	127 (11.4)	168 (13.1)	189 (17.2)	< 0.0001
Intrapartum-related complications	84 (12.1)	163 (14.6)	199 (15.6)	149 (13.6)	0.04 ^b^
Meconium aspiration syndrome	5 (0.7)	18 (1.6)	24 (1.9)	20 (1.8)	0.09
Respiratory distress	67 (9.7)	177 (15.9)	302 (23.6)	98 (8.9)	< 0.0001 ^b^
Other infections	87 (12.6)	172 (15.4)	127 (9.9)	104 (9.5)	0.0003
Malformations	32 (4.6)	23 (2.1)	37 (2.9)	37 (3.4)	0.01 ^b^

Data expressed as n (%) or ^a^ median (IQR). ^b^ Quadratic curve over time

## Discussion

Our findings indicated some neonatal characteristics (outborn, lower weight and age at admission) and a subset of diagnoses (hypothermia at admission, late-onset sepsis, low birth weight, intrapartum-related complications, MAS, respiratory distress, malformations and other infections) as risk factors for mortality after admission to the special care unit of a low-resource setting.

While neonatal mortality in high-resource countries is usually due to unpreventable causes, the majority of neonatal deaths in low-resource areas occur from preventable and treatable diseases, including intrapartum-related complications, prematurity and infections [9, 23].

In the last two decades, Ethiopia has succeeded in reducing neonatal mortality rate from 49.5 deaths to 28.1 deaths per 1000 live births thanks to many efforts from the government and other stakeholders [24]. Despite the implementation of the National Child Survival Strategy (2005–2015) [25], Ethiopia has still one of the highest neonatal mortality rates worldwide [6]. Investigation of mortality among neonates admitted to the special care unit in a low-resource setting is an important step for planning appropriate interventions [9, 23].

The magnitude of neonatal mortality was 17% among admissions to the special care unit of the St. Luke Wolisso Hospital during 2014–2017, which laid in the mortality range of previous studies in Ethiopia [11–13, 26]. The majority of deaths (84%) occurred during the first week of admission - in agreement with previous studies [12, 26] - with low birth weight, intrapartum-related complications, malformation and respiratory distress representing a heavy burden on neonatal mortality. These findings suggest the need for further efforts in improving labour, intrapartum and immediate postnatal newborn care practices [11].

Our analysis of risk factors of mortality confirmed the role of low birth weight [9], which is known to contribute to the largest number of both admissions and deaths in low-resource settings. Thermal care and appropriate feeding play an important role in these neonates, thus prevention and treatment of hypothermia (i.e. kangaroo mother care) and the promotion of early and exclusive breastfeeding are warranted [27]. Despite the very high mortality among ELBW and VLBW infants, their limited occurrence along with constrains for their treatment in low-resource settings suggested that efforts should target neonates with birth weight 1500–2500 g. In addition, other comorbidities (intrapartum-related complications, late-onset sepsis, MAS, respiratory distress) and malformations were also associated with increased risk of neonatal mortality. All these factors can be both preventable (through appropriate antenatal and perinatal care) and cared for (with available skills and equipment) in the special care unit [9]. Quality improvement initiatives to reduce neonatal mortality should focus on strengthening the continuum of care including fetal, intrapartum and postnatal phases [9].

Outborn neonates and those with moderate hypothermia at admission were also identified as subjects at high risk of mortality. This is noteworthy since half of admissions were outborn, which mirrors the geographical distribution of population in Ethiopia, where over 80% of people resides in the rural part of the country [24]. Of note, our data suggested that the increased mortality risk in outborn neonates was not due to their temperature at admission.

About half of neonates were hypothermic at admission, thus underlying the importance of thermal control during the postnatal period [28]. Neonatal hypothermia is common in both health facilities and homes, even in tropical environments. While hypothermia is not often considered a direct cause of death, it contributes to a substantial proportion of neonatal mortality, mostly as a comorbidity of severe neonatal infections, preterm birth, and intrapartum-related complications [28, 29]. Of note, the surprising high proportion of hypothermia among inborn neonates calls for urgent actions for preventing thermal losses immediately after delivery. In low-resource settings, such condition is likely to persist in the days following birth, with negative impact on prognosis [30].

During the study period, the implementation of quality improvement interventions (education on neonatal resuscitation, courses on postnatal management, and the introduction of an on-call doctor for high-risk deliveries) could contribute to explain the mortality reduction in 2014–2017 [31]. In addition, we observed different trends over time of factors associated with neonatal mortality. While these data provide useful information about the changing characteristics of admitted neonates, the interpretation goes beyond the scope of the present study and should rely on a longer time span.

During the study, problems in documentation emerged when retrieving data from hospital charts. In fact, information on important prognostic indicators (such as temperature at admission and place/mode of delivery) was missing in 15–30% of the records. The underreporting of such indicators highlights the need for enhancing the awareness of the importance of including those measurements among routine care [32].

This study has some limitations. First, it is a single-center study thus generalizability is limited to similar settings. Second, the retrospective data collection limited the quality and completeness of available information. Third, the diagnosis was mostly based on clinical examination due to the limited availability of laboratory and instrumental equipment.

Our study adds information about risk for mortality among neonates admitted to a special care unit in Ethiopia, where available literature on risk factors for neonatal mortality is limited [11–13, 27]. Our findings confirm that the majority of neonatal deaths seemed to be associated with preventable and treatable conditions [14]. Thus, improvements of referral system, ante- and perinatal care, and postnatal management are warranted to reduce neonatal mortality [11, 15]. Of note, education to health care givers, audits and continuous feedback should be implemented to improve quality and completeness of documentation.

Our findings also contribute to feed up an on-going systematic review which aims at filling the gap in understanding burden and risk factors of neonatal mortality in Ethiopia [14]. The summary of the available evidence will inform health policy makers and stakeholders about which factors should be targeted to reduce neonatal mortality in Ethiopia [14].

## Conclusions

Our findings showed that neonatal mortality was associated with admission at early age, low birthweight, being outborn, late-onset sepsis, intrapartum-related complications, meconium aspiration syndrome, respiratory distress, infections, malformations and hypothermia. Education on neonatal resuscitation and postnatal management, and the introduction of an on-call doctor for high-risk deliveries might have contributed to the reduction in neonatal mortality over time.

## Data Availability

The material of the current study is be available from the corresponding author on reasonable request.

## Abbreviations

CI: Confidence interval
ELBW: Extremely low birthweight
LBW: Low birthweight
MAS: Meconium aspiration syndrome
OR: Odds ratio
VLBW: Very low birthweight
